# Circadian variation on the onset of acute ST segment elevation myocardial infarction in diabetic subjects

**DOI:** 10.4103/0975-3583.59981

**Published:** 2010

**Authors:** Jing Li, Qi Hua, Lin Pi, Jing Tan, Boyu Li

**Affiliations:** * *Heart Center; Beijing Xuanwu Hospital Affiliated Capital Medical University, Beijing, PRC*; # Heart Center; Beijing Chui Yangliu Hospital, Beijing, PRC

**Keywords:** *myocardial infarction*, *circadian variation*, *diabetes*

## Abstract

**Background—:**

Previous studies have shown that there is a circadian variation in the incidence of acute myocardial infarction. The aim of this study is to examine the circadian rhythms of acute myocardial infarction in patients with type 2 diabetes.

**Methods and Results—:**

1016 consecutive patients admitted to a coronary care unit with acute ST elevation myocardial infarction were studied from January 2004 to December 2008. All patients were divided into two groups according to with or without diabetes. Admission rates were calculated according to the 6-hour interval of the day (circadian rhythm). The data were analyzed for variations within subgroups. In diabetic group, number of patients in the first to fourth quarters was 38, 45, 43, 46 respectively (NS). The corresponding figures for the controls were 174, 295, 183, 192 (P<0.01). The difference between the two groups was significant (P<0.02).

**Conclusion—:**

There is no a significant circadian variation in the onset of acute myocardial infarction in diabetic subjects.

## INTRODUCTION

Circadian variation of the onset of acute myocardial infarction (AMI) has been reported by some studies and may carry important pathophysiologic implications. Some studies show an increased onset in the morning between 6:00 AM and 12:00 noon. Furthermore, a secondary peak in the late evening has also been reported in some studies.^1^

Diabetes neuropathy occurs in approximately 16~50% of individuals with long-standing type 1 and type 2 diabetes. It may manifest as polyneuropathy, mononeuropathy^2^ and/ or autonomic neuropathy. Diabetic neuropathy may lead to loss of the normal circadian pattern of autonomic nervous system activity^3^, and altered normal circadian variation in some physiological process.^4^ Zarich et al report that time of onset of ischemia in diabetic patients follows a circadian distribution, with a peak incidence in the morning hours, suggesting that alterations in sympathovagal balance may have an effect on the circadian pattern of cardiovascular events.^5^ However, there are conflicting reports regarding circadian variation on the onset of AMI among patients with diabetes. To determine whether diabetes has an impact on the circadian variation of AMI onset, we investigate the circadian pattern in diabetic patients and compare with the controls.

### Design and methods

### Study Population

Consecutive patients admitted to the cardiac care unit (CCU) of Xuanwu hospital with ST segment elevation myocardial infarction (STEMI) between January 2004 and December 2008 were enrolled. Inclusion criteria were: age greater than or equal to 18 and diagnosis of STEMI. STEMI was diagnosed if two of the following criteria were present: (1) the presence of ischemic pain or other symptoms lasting ≥30 minutes; (2) ST-segment elevation of ≥ 2 mm in at least 2 contiguous precordial leads, ST-segment elevation of ≥1mm in at least 2 inferior leads (II, III, or aVF), or new left bundlebranch block; (3) an increase in serum MB isoenzyme of creatine kinase(CKMB) to more than twice the upper limit of normal. Exclusion criteria: (1) myocardial infarction occurring after invasive coronary artery procedures such as percutaneous coronary intervention(PCI) or coronary artery bypass grafting(CABG ; (2) history of myocardial infarction; (3) causes of myocardial infarction without coronary atherosclerosis.

All subjects were assigned into two groups according to with or without diabetes.

The diagnosis of type 2 diabetes mellitus was based on history of treatment with hypoglycemic agents and/or fasting blood glucose >126 mg/dl.

We divided the day in into four 6-h intervals from 0:00 to 5:59, 6:00 to 11:59, 12:00 to 17:59, and 18:00 to 23:59 and calculated number of patients in each interval.

### Data collection

For each patient enrolled, the structured data form was completed by experienced cardiologists within 72 hours after admission.

Clinical features were obtained from coronary care unit admission logs and other medical documentation, including: (1) baseline data: age, gender, systolic and diastolic blood pressure, heart rate, smoking, history of disease (diabetes, hypertension, cerebral vascular disease). Duration from onset to emergency room was calculated. (2) laboratory examination: white cell count (WBC), total cholestrol, peak of MB isoenzyme of creatine kinase, serum potassium, blood glucose, serum creatinine (CRE). Fasting blood samples for laboratory measurements were obtained early in the morning after overnight fasting within 24h of admission. Left ventricle ejection fraction (LVEF) was measured by ultrasonic cardiogram (UCG) within 24 hours of admission. (3) Clinical outcome: Killip Class, in-hospital mortality.

### Statistical analysis

The hypothesis that the number of diabetes patients with infarction during each time interval was equal to an expected frequency was tested using a χ2 goodness of fit analysis. The χ2 test was used to assess the significance of circadian variation between the two groups.

## RESULTS

### 1. Baseline characteristics

Among 1016 subjects enrolled in this study, 172 were diagnosed as diabetes. Compared with the control group, diabetes patients were elder and more female. There was no significant difference on level of serum creatinine and left ventricular ejection fraction between two groups. Diabetes patients had more comorbidities and worse in-hospital outcome. Proportion of patients taking β adrenergic receptor blockers before onset of MI was similar between two groups (table 1).

**Table 1:** Comparison of baseline characteristics of patients between two groups

	Diabetes group	Control group	*P* value
N	172(16.9%)	844(83.1%)	
Age(year)	65.3±10.5	62.4±12.4	0.001
Men(%)	105(61.0)	632(74.9)	<0.001
Women (%)	67(39.0)	212(25.1)	<0.001
Time(h)	5.0	5.0	0.756
Heart rate (bpm)	84.2±20.7	77.9±17.5	<0.001
Systolic pressure(mmHg)	133.6±28.9	132.8±44.6	0.833
Diastolic pressure(mmHg)	80.4±17.0	80.6±16.5	0.419
Killip Class(%)	0.006		
I	82(48.0)	512(62.2)	
II	59(34.5)	225(26.8)	
III	17(9.9)	62(7.4)	
IV	13(7.6)	29(3.5)	
WBC(G/L)	10.6±3.8	10.9±5.8	0.801
Peak of CKMB(IU/L)	121.0	144.0	0.041
Cholesterol(mg/dl)	191.4±47.8	186.6±44.7	0.222
Serum creatinine(mg/dl)	1.3±0.5	1.2±0.9	0.123
Serum potassium (mmol/L)	4.3±0.7	4.1±1.5	0.688
Ejection fraction(%)	55.9±11.0	56.0±10.6	0.936
Hypertension(%)	111(64.5%)	367(43.5%)	<0.001
Smoker(%)	49(28.5%)	448(53.1%)	<0.001
Cerebral vascular disease	39(22.8%)	88(10.6%)	0.001
Death(%)	32(18.6%)	57(6.7%)	<0.001
Prior β blocker	29(17.0%)	34(19.6%)	0.0

Data are presented as means±SD, median or cases(percentages) of patient. WBC, white blood corpuscle; CKMB, MB isoenzyme of creatine kinase

### 2. Circadian variation

The 6-h interval of AMI onset was shown in Table 2. There was no circadian variation in the incidence of acute myocardial infarction in diabetic subjects. The circadian variation was significant in the controls (P<0.01). The peak incidence occurred in the second quarter (6 am to 12 noon), this was statistically higher than the average incidence in the remainder of the day (P<0.01). The difference between the circadian pattern of diabetic patients and controls was highly statistically significant (P<0.02).

**Table 2:** distribution of 6-hour interval

	0:00~5:59	6:00~11:59	12:00~17:59	18:00~23:59
diabetes group	38(22.2%)	45(26.1%)	43(24.9%)	46(26.8%)
control group	174(20.6%)	295(34.9%)	183(21.7%)	192(22.8%)

Data are presented as case s(percentages) of patient

## DISCUSSION

Circadian variation has been demonstrated in several types of acute cardiovascular disease, including acute myocardial infarction, sudden cardiac death, silent ambulatory ischemia, and thrombotic stroke. The morning peak of incidence of AMI is related to some known daily rhythms. It is well known that a surge in sympathetic activity and vagal withdrawal occurs after waking accompanying by increase of plasma levels of catecholamine, renin and cortisol (morning peak approximately 6:00 AM, decreasing but still high until noon). As a result, higher level of heart rate, blood pressure, coronary vascular tone, platelet aggregability and a lower level of fibrinolytic activity are observed during the early morning hours. These changes may increase shear forces in the coronary arterial bed, thus promoting plaque disruption and causing unstable angina and AMI. Also, a morning increase in platelet reactivity may make a thrombus more likely to grow and cause symptoms.

A study^6^ including 4,796 patients firstly showed that circadian variation of the onset of acute myocardial infarction altered with specific clinical characteristics such as diabetes, smoking, heart failure, non Q wave infarction, taking beta-blocking drugs. Some study had confirmed this finding,^7^ but other study showed conflicting reports. They failed to demonstrate such a variation in the circadian pattern in the onset of AMI[8] among patients with diabetes. Our study showed there was no a significant morning peak in patients with diabetes but was obvious in control group (6 am to 12 noon).

The mechanism of change of circadian pattern in the onset of AMI among diabetic subjects is unclear. It can be related to the blunting of diurnal variation in physiological variables. Autonomic nervous system plays an important role in determining the circadian pattern of cardiovascular events. It suggests that in patients with diabetes, abnormalities in the circadian rhythm of autonomic tone may be responsible for the altered temporal onset of cardiovascular events.^9^~^12^ One study^13^ found that 25.3% of patients with type 1 diabetes and 34.3% of patients with type 2 diabetes had abnormal findings in more than two of six autonomic function tests. Another study observed prevalence rate of autonomic neuropathy for individuals with type 1 diabetes was 16.6%.^14^

As a result of autonomic neuropathy, the rhythm of sympathovagal balance is significantly attenuated in patients with diabetes compared with those without diabetes. The morning rise in platelet aggregability has been reported to be lost in diabetic patients by some investigators. Plasminogen activator inhibitor 1 and von Willebrand factor show no circadian variation in patients with diabetes.^15^ Diabetic subjects also show diminished circadian variation in blood pressure.^16^ Yamamoto reported patients with diabetes and symptomatic autonomic neuropathy had markedly impaired heart rate variability.^17^

However, there were also conflicted reports. Behar showed the preponderance of the morning peak persisted in patients with diabetes mellitus.^18^ Jamal reported circadian morning peak of AMI symptom onset existed in patient with history of type 2 diabetes less than 5 years, but it was attenuated in patients with type 1 diabetes or type 2 diabetes for 5 or more years.^19^ In this study, we demonstrated disappear of morning peak of onset of AMI was not associated with duration of diabetes. In fact, while autonomic dysfunction is an established complication of diabetes and clinical symptoms of autonomic neuropathy generally do not occur until long after the onset of diabetes, but impaired autonomic function is often detected at the time of diabetes diagnosis. Subclinical autonomic dysfunction can, however, occur within a year of diagnosis in type 2 diabetes patients and within two years in type 1 diabetes patients. This suggests that autonomic dysfunction may be present after a relatively brief exposure to hyperglycemia or perhaps even in the clinically normal range of glucose.^20^–^21^ Data in our study which indicates that resting heart rate of diabetic subjects is higher than non diabetics, maybe support this opinion indirectly, because resting tachycardia is a marker of autonomic dysfunction.

The lack of circadian variation in the onset of acute myocardial infarction may have therapeutic implications. As cardio-protective medication has been shown to exert its effect mainly by diminishing the morning peak in acute myocardial infarction, the optimal timing of such medication may differ in diabetic subjects from their non-diabetic counterparts.^22^
